# Description of a second South American species in the Malagasy earwig genus *Mesodiplatys* from a cave habitat, with notes on the definition of Haplodiplatyidae (Insecta, Dermaptera)

**DOI:** 10.3897/zookeys.790.27193

**Published:** 2018-10-15

**Authors:** Yoshitaka Kamimura, Rodrigo L. Ferreira

**Affiliations:** 1 Department of Biology, Keio University, 4-1-1 Hiyoshi, Yokohama 223-8521, Japan Keio University Yokohama Japan; 2 Center of Studies in Subterranean Biology, Biology Department, Federal University of Lavras, CEP 37200-000 Lavras (MG), Brazil Federal University of Lavras Minas Gerais Brazil

**Keywords:** Brazil, cave insects, *
Haplodiplatys
*, Madagascar, *Mesodiplatysfalcifer* sp. n.

## Abstract

The genus *Mesodiplatys* (Dermaptera: Diplatyidae) comprises eight species from Madagascar and one species from Peru. Based on a sample collected from a cave in Brazil, a new species of this genus, *Mesodiplatysfalcifer* Kamimura, **sp. n.**, is described as the second species from South America. Based on a reexamination of the holotype of *Mesodiplatysinsularis*, a revised key to *Mesodiplatys* species is provided. The definitions of the genera *Mesodiplatys* and *Haplodiplatys* and the family Haplodiplatyidae are also reconsidered.

## Introduction

Diplatyidae (*sensu*Sakai 1996, including *Haplodiplatys* and Cylindrogastrinae) is an earwig family comprising approximately 150 described species. This family shows typical Gondwanan distribution (Popham 2000), possessing many plesiomorphic characteristics (Haas 1995; Haas and Kukalová-Peck 2001; Haas and Klass 2003; Klass 2003). Among diplatyids, the members of the genus *Haplodiplatys* Hincks, 1955 (type species; *Haplodiplatysniger* Hincks, 1955) are considered to be among the earliest offshoots in the suborder Neodermaptera (i.e., extant earwigs: Haas 1995; Popham 2000; Haas and Kukalová-Peck 2001; Haas and Klass 2003). Recently, Engel et al. (2017) treated *Haplodiplatys* as the sole genus of the family Haplodiplatyidae, and we follow this approach in the present study.

The genus *Mesodiplatys* was originally proposed as a subgenus of *Haplodiplatys* with the type species *Diplatysnana* Burr, 1914 (Steinmann 1986b). Although the parameres (= external parameres or metaparamers) are relatively simple (lacking teeth, projections, and branching) as in other *Haplodiplatys* spp., Anisyutkin (2014) treated *Mesodiplatys* as a separate Diplatyidae genus based on many differences in external morphology, including the differentiation of the head into frontal and occipital regions.

The distribution of this genus is enigmatic: eight species are known from Madagascar and one species from Peru. Anisyutkin (2014) proposed three hypotheses to explain this disjunct distribution. First, the observed similarities in genital morphology between Malagasy and South American *Mesodiplatys* species evolved in parallel. Second, this genus displays Gondwanan distribution and African species remain undiscovered or have gone extinct. Third, the common ancestor of *Mesodiplatys* originated in Madagascar and then expanded its distribution to South America (or vice versa). Case (2002) proposed a land bridge (the Gunnerus Ridge) between Madagascar and Antarctica in the middle to late Cretaceous. Although recent estimates based on geological and geophysical data do not support the presence of such a causeway (Ali and Krause 2011), similar disjunct distributions in Madagascar and South America are known for several animal groups, including some boid snakes (Noonan and Chippindale 2006a), iguanids (pleurodont iguanians: Noonan and Chippindale 2006b), and podocnemidid turtles (Noonan and Chippindale 2006b), suggesting past direct bioconnections between these regions.

To explore these possibilities, extensive taxonomic surveys in South American and African regions are needed. In the current study, we report and describe in detail the second *Mesodiplatys* species from South America based on a male sample collected from a Brazilian cave. For comparison, the external morphology is re-described based on the *M.insularis* (Borelli, 1932) type specimens. A contemporary key is provided for males of *Mesodiplatys* spp. Based on our results, we briefly discuss the definitions of the genera, *Mesodiplatys* (Diplatyidae) and *Haplodiplatys* (Haplodiplatyidae).

## Materials and methods

The material examined in this study was collected from a cave in Brazil in 2017, and preserved in 70% ethanol. After removal of the genitalia, the specimen was mounted on cardboard using fish glue and allowed to dry. The genitalia were examined under a BX53 differential interference contrast (DIC) microscope (Olympus, Tokyo, Japan), mounted in Euparal (Waldeck GmbH and Co. KG, Münster, Germany) between two coverslips, and attached to the pin of the specimen. Based on photographs taken under the DIC microscope and a S8-APO stereo microscope (Leica, Wetzlar, Germany), we created a digital composition of selected in-focus parts of each image using the Combine ZM image-stacking software (Hadley 2008).

For comparison, external morphologies of the holotype (male) and a paratype (female) of *M.insularis* deposited in the Zoological Museum (**ZMH**), part of the Centrum für Naturkunde (**CeNak**) at the Universität Hamburg, were examined based on photographs provided by CeNak staff.

The type of the newly described species was deposited in the Subterranean Invertebrate Collection of Lavras (**ISLA**), at the Universidade Federal de Lavras (**UFLA**), Lavras, Brazil.

We followed Kamimura and Ferreira (2017) in the suprageneric classification of the infraorder Protodermaptera. The terminology of Kamimura (2014) was used to describe male genital structures.

## Taxonomy

### Order DERMAPTERA de Geer, 1773

#### Infraorder PROTODERMAPTERA Zacher, 1910

##### Family DIPLATYIDAE Verhoeff, 1902

###### Genus *Mesodiplatys* Steinmann, 1986

####### Mesodiplatys falcifer

Taxon classificationAnimaliaDermapteraDiplatyidae

Kamimura sp. n.

http://zoobank.org/2BE92C3E-D821-4593-BF57-11D44A027CE5

Figs 1–6 , 7–14

######## Material examined.

Holotype ♂, ‘Lapa dos Peixes II | (UTM 612750, 8971635) | Carinhanha municipality,| Bahia, Brasil’, ‘ISLA 46682’, ‘15.x.2017 | Coleta Geral (general sampling), Ferreira R.L. leg.’, ‘HOLOTYPE (male) | *Mesodiplatysfalcifer* | sp. nov. | Det. Y. Kamimura 2018’.

######## Diagnosis.

*Mesodiplatysfalcifer* sp. n. is a small-sized species with a slender abdomen and simple forceps. This species differs all other species of *Mesodiplatys* with the combination of the following characters: the sickle-shaped sclerite in the penis lobe; notably short parameres lacking dentiform curvature near base on inner margin and its articulation with main body of genitalia perpendicular to its anterior-posterior axis; notably large eyes; and uniformly pale pronotum and darker tegmina.

######## Description.

***Male*** (holotype, Figure 7). Measurements are shown in Table 1. Body color generally amber except for 3^rd^ antennal segment and beyond, distal third of femur, basal half of tibia, tegmina (except for region around scutellum), fustis of wings, dorsal side of 7^th^ abdominal segments and beyond, and lateral sides of 6^th^ abdominal segment dark brown. Head and 1^st^ and 2^nd^ antennal segments black. Forceps reddish brown. Abdomen and forceps densely pubescent.

**Table 1.** Measurements of the male holotype of *Mesodiplatysfalcifer* sp. n. (mm) in comparison with those of the South American congener *M.venado* (Anisyutkin, 2014).

	*M.falcifer* sp. n. (A)	*M.venado* (B)	Ratio (A/B)
***Length***			
Body without forceps	8	-	-
Head	1.1	1.5	0.73
Pronotum	0.87	1.0	0.87
Tegmen	2.3	2.9	0.79
Fore/mid/hind femur	1.5/1.8/2.2	1.6/2.0/2.2	0.94/0.90/1.0
Fore/mid/hind tibia	1.5/1.5/2.0	1.7/1.8/2.2	0.88/0.83/0.90
Forceps	1.3	1.8	0.72
***Width***			
Head	1.2	1.7	0.71
Pronorum	0.76	1.1	0.69
Tegmen	0.66	0.9	0.73
Ultimate tergite	0.80	1.2	0.67

**Figures 1–6.**
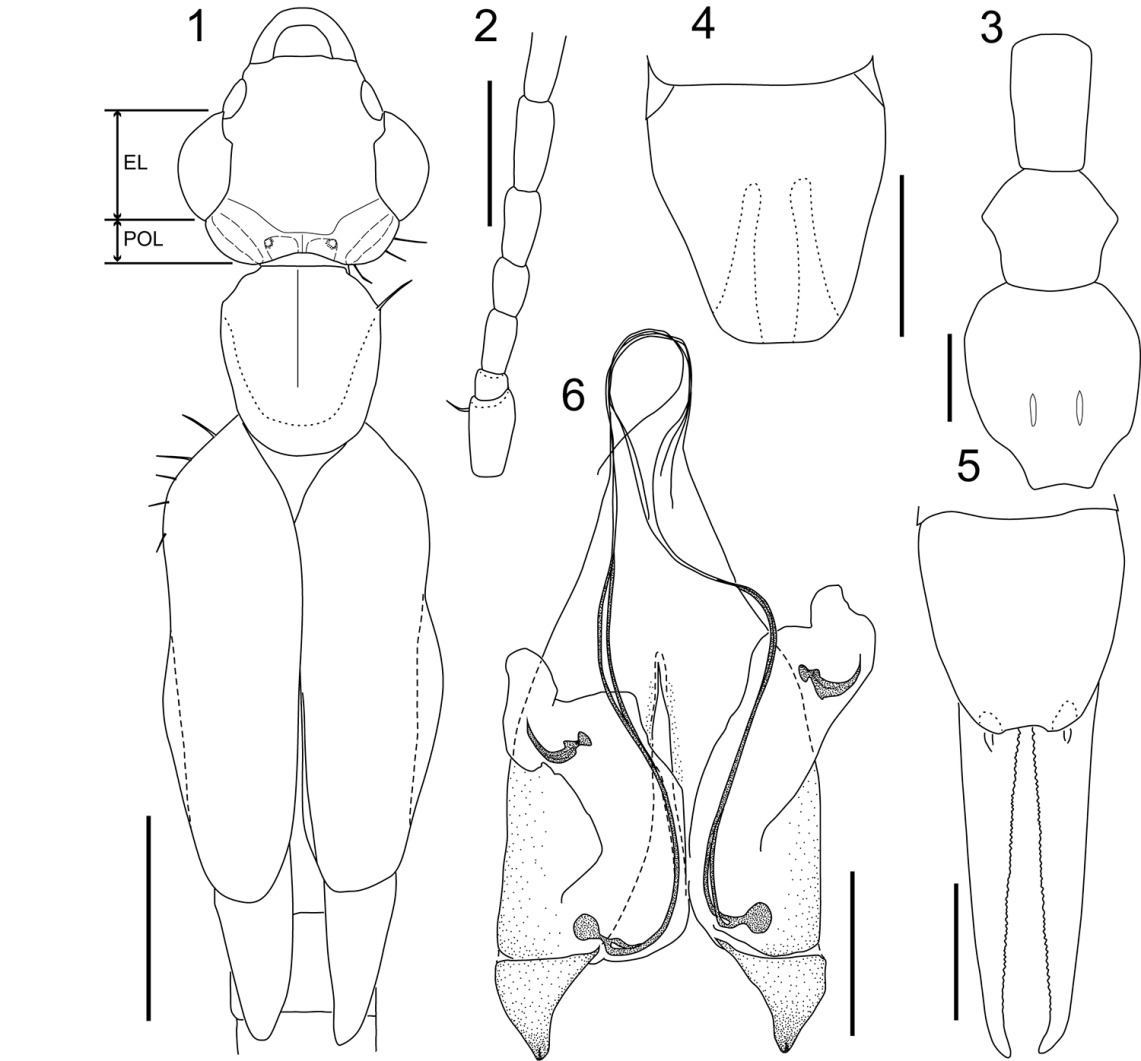
*Mesodiplatysfalcifer* sp. n. (male, holotype): **1** head, thorax, and proximal part of abdomen **2** proximal part of left antenna **3** thoracic sternites **4** penultimate abdominal sternite **5** ultimate abdominal tergite and forceps **6** genitalia. Abbreviations: EL, eye length; POL, post-ocular length. Scale bars: 1 mm (**1)**; 0.5 mm (**2–5)**; 250 μm (**6).**

Head (Figs 1, 8) slightly longer than width, widest in eye region; frons tumid but weakly depressed at apex; occiput strongly and widely depressed; transverse and median sutures visible but not conspicuous; posterior margin strongly emarginated in middle with a pair of semi-oval tubercles lateral to median suture, of which outer-anterior angle protrudes dorsally as small papilla; post-ocular carina conspicuous as oblong swelling; lateral margins of post-ocular region bordered by strong bristles. Eyes conspicuously prominent, eye length 2.5 times the post-ocular length (EL/POL, measured along anterior-posterior axis as shown in Figure 1; Table 2). Antennae (Figs 2, 8) comprise 19 segments: segment I stout, expanded apically, length almost half of distance between antennal bases; segment II shorter than width; segment III twice as long as width; segment IV 1.5 times longer than width; segment V almost as long as III; remaining segments gradually lengthening. Pronotum (Figs 1, 8) slightly longer than broad; anterior margin almost straight; humeral angles strong; sides weakly narrow posteriorly; posterior margin broadly rounded; median sulcus visible but not distinct; prozona weakly raised. Tegmina (Figs 1, 8) well developed, approx. twice as wide as pronotum, 2.5 times as long as pronotum, broad triangular scutellum visible. Mesonotum with well-developed spiny ridge (a component of tegmina locking device sensu Haas 1995). Wings (Figure 1) well developed. Legs slender, notably long (Table 1); hind tarsi with segments I and II 3–3.5 times as long as III, claw with small arolium. Prosternum elongated, semi-rectangular, not constricted at middle; mesosternum hexagonal; metasternum semi-oval, narrowing posteriorly, posterior margin emarginated (Figure 3). Abdomen long, cylindrical, segments VIII and IX slightly expanded. Penultimate sternite (= sternite IX) elongated (Figs 4, 11), posterior margin almost truncate with rounded angles, regions at base of forceps weakly raised. Ultimate tergite (= tergite X) moderately inflated (Figs 5, 10), semi-oval, narrowing and sloping posteriorly. Forceps (cerci; Figs 5, 10, 11) slender, almost straight, densely pubescent especially on inner margins, tapering and weakly curving inward apically, inner margin with small tooth. Genitalia (Figs 6, 12–14), virga consists of bifurcated thin tubes, approx. three times as long as penis lobe, and 1.2 times as long as main part of genitalia (from base to distal end of paramere), spherical vesicle at base followed by short common duct; penis lobe also encloses sickle-shaped sclerite in characteristic swelling; parameres short, simple, triangular, distal part curving dorsally with many short spines near apex.

**Table 2.** Eye length (EL) relative to post-ocular length (POL) in male *Mesodiplatys* species. Measurements are as shown in Figure 1.

	Ratio (EL/POL)	Source image used for measurement
**South American species**		
* M. venado *	2.07	Fig. 1 (Anisyutkin 2014)
*M.falcifer* sp. n.	2.50	Fig. 1 (this study)
**Malagasy species**		
* M. nanus *	1.99	Fig. 7 (Brindle 1966)
* M. insularis *	1.61	Fig. 16 (this study)
* M. olsufiewi *	0.69	Fig. 1 (Brindle 1967)
* M. longicornis *	1.35	Fig. 16 (Hincks 1955)
* M. gracillimus *	1.75	Fig. 4 (Brindle 1966)
* M. mucronatus *	1.35	Fig. 4 (Brindle 1969)
* M. major *	1.27	Fig. 1 (Brindle 1966)
* M. raharizoninai *	1.90	Fig. 5 (Brindle 1969)

**Figures 7–14.**
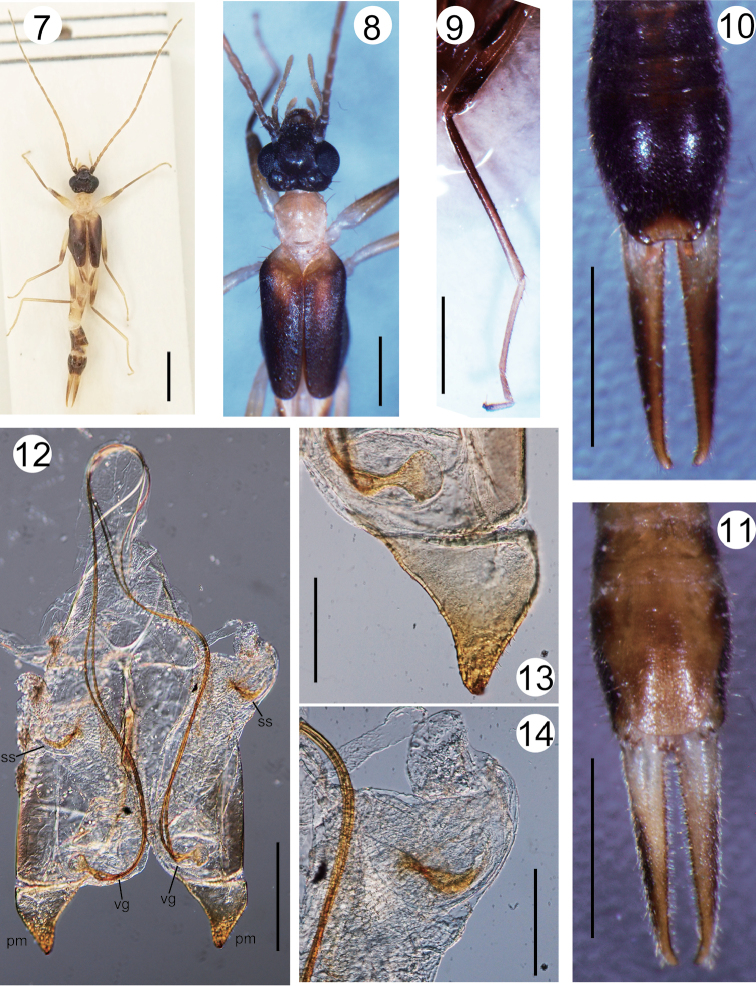
*Mesodiplatysfalcifer* sp. n. (male, holotype): **7** habitus **8** head, thorax, and tegmina **9** right hind leg **10** tergites of post-abdomen and forceps **11** sternites of post-abdomen and forceps **12** general view of genitalia **13** right parameres and basal part of virga **14** sickle-shaped sclerite in right penis lobe (Fig. 14). Abbreviations: pm, paramere; ss, sickle-shaped sclerite; vg, virga. Scale bars: 2 mm (**7)**; 1 mm (**8–11**); 250 μm (**12)** ; 100 μm (**13, 14)**.

***Female.*** Unknown.

######## Remarks.

*Mesodiplatysfalcifer* sp. n. is apparently allied to *M.venado* Anisyutkin, 2014 recorded from Peru. However, in addition to the characteristic sickle-shaped sclerite in the penis lobe, the former species is distinguished from the latter by possession of triangular parameres, penultimate sternite with truncated posterior margin, and pale coloration of the pronotum. This new species is also differentiated from all Malagasy members of the genus with the combination of the following characters: sickle-shaped, distinct sclerite in penis lobe; notably short parameres lacking dentiform curvature near base on inner margin and its articulation with main body of genitalia perpendicular to its anterior-posterior axis; eyes notably longer than POL (Table 2); and uniformly pale pronotum with darker tegmina.

**Figures 15–20.**
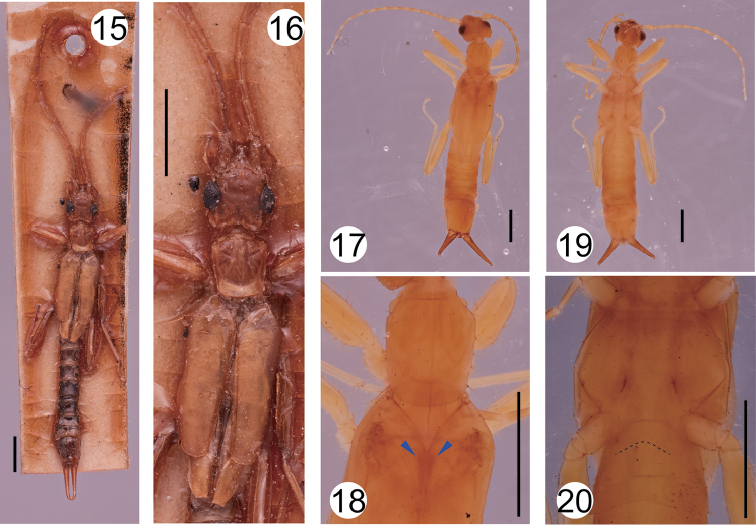
**15, 16***Mesodiplatysinsularis* (Borelli, 1932) (male, holotype): **15** habitus **16** head, thorax, and proximal part of abdomen. **17–20***Mesodiplatysinsularis* (female, paratype): **17** habitus from above **18** thorax and proximal part of tegmina **19** habitus from below **20** metasternum. Blue arrowheads in **18** indicate spiny ridges. Broken line in **20** indicates the posterior margin of the metasternum. Scale bar: 2 mm.

Previous authors have noted that the male of the Malagasy species *M.insularis* (Borelli, 1932) has conspicuously large eyes, twice the POL in length (Hincks 1955; Brindle 1966, 1969). However, our reexamination of the holotype (male: Figure 15) at ZMH revealed that its eye length is only 1.6 times the POL (Figure 16), shorter than those of the two South American species (2.07–2.50) and some Malagasy species including *M.nanus* (Burr, 1914) and *M.raharizoninai* (Brindle, 1966) (Table 2). The genitalia of this species have not been illustrated. Although Borelli (1932) briefly described their characteristics including simple parameres and very long virga branches, the holotype does not include genitalia, which were likely removed from the main body. We were unable to locate any other slide-mounted materials derived from this specimen within the ZMH collection.

Thoracic traits, other than the shapes of the pronotum and tegmen, have not been described in detail for any Malagasy members of *Mesodiplatys* (Burr 1904, 1911, 1914; Borelli 1932a,b; Hincks 1953, 1955, 1957; Brindle 1966, 1967, 1969; Steinmann 1974, 1986a,b). In the single paratype female of this species (Figs 17, 19), which has been preserved in ethanol, well-developed spiny ridges are visible through the tegmina (Figure 18). The morphologies of the thoracic sternites of this paratype are essentially similar to those of *M.falcifer* sp. n., including conspicuous emargination at the middle of the posterior margin of the metasternum (Figure 20). As another candidate member of *Mesodiplatys*, *Diplatysviator* Burr, 1904 has been reported from Madagascar. However, this species was described based only on a broken and discolored female specimen. Hincks (1955) treated *Diplatyshova* Burr, 1914, which was also described from a single male collected in Madagascar, as a junior synonym of *D.viator*. Although Hincks found a possible female specimen of this species in Burr’s collection, he failed to trace the male holotype, leaving the position of this species doubtful.

**Key to known *Mesodiplatys* species (males only)**

**Table:** 

1	Both pronotum and tegmina uniformly yellow or yellowish brown	**2**
–	Pronotum and/or tegmina uniformly blackish or dark brown, or pale brown with darker markings	**6**
2	Small species, 9 mm or smaller in total length (including forceps)	**3**
–	Large species, 14 mm or larger in total length (including forceps)	**4**
3	Virga short, not exceeding base of genitalia when in repose. Mucro at distal apex of parameres. No conspicuous denticulated sclerite in the penis lobe	***Mesodiplatysmucronatus* (Hincks, 1957)**
–	Virga relatively long, apparently exceeding base of genitalia. No mucro at distal apex of parameres. Rectangular denticulated sclerite in the penis lobe	***Mesodiplatysnanus* (Burr, 1914)**
4	Penultimate sternite truncate, not sinuated or notched at the middle of the posterior margin	***Mesodiplatysraharizoninai* (Brindle, 1966)**
–	Posterior margin of penultimate sternite weakly sinuated at the middle, or with conspicuous emargination	**5**
5	Pronotum more than 1.5 times as long as broad	***Mesodiplatyslongicornis* (Hincks, 1955)**
–	Pronotum almost quadrate, slightly longer than broad (Fig. 16)	***Mesodiplatysinsularis* (Borelli, 1932)**
6	Eyes very small, shorter than post-ocular length. Body shiny and black	***Mesodiplatysolsufiewi* (Borelli, 1932)**
–	Eyes large, longer than post-ocular length. Body not shiny and black	**7**
7	Tegmina blackish, with conspicuous yellow marking at center	***Mesodiplatysgracillimus* (Hincks, 1957)**
–	Tegmina not blackish. If blackish, then without conspicuous yellow marking at center	**8**
8	Minute dentiform curvature near base on inner margin of parameres. Forceps macrolabic, asymmetrical, with left branch more strongly curved than right	***Mesodiplatysmajor* (Brindle, 1966)**
–	Inner margin of parameres uniformly rounded without dentiform curvature. Forceps symmetrical	**9**
9	Penis lobe lacks accessory sclerites. Posterior margin of penultimate sternite with conspicuous emargination at the middle. Pronotum, dark brownish. Paramere obtuse, thumb-shaped	***Mesodiplatysvenado* Anisyutkin, 2014**
–	Sickle-shaped sclerite in penis lobe. Penultimate sternite truncate, not sinuated at the middle of posterior margin. Pronotum pale. Parameres subtriangular	***Mesodiplatysfalcifer* Kamimura, sp. n.**

######## Etymology.

The species epithet refers to the sickle- or falx-shaped sclerite in the penis lobe, which is characteristic to this new species among the species of the genus *Mesodiplatys* known to date.

######## Distribution.

Bahia, Brazil.

## Discussion

### Association with caves

This is the first report of a *Mesodiplatys* species from a cave habitat. The *Mesodiplatysfalcifer* sp. n. specimen was collected in a cave (Lapa dos Peixes II) located in a region known as Serra do Ramalho, which is characterized by extensive limestone outcrops extending for dozens of kilometers. Lapa dos Peixes II is the cave furthest downstream among a four-cave system comprising more than 23 km of galleries. This cave represents a horizontal length of 2,100 m, and its main conduit contains some submerged areas (lakes). The adult specimen was found freely walking on the surface of a speleothem, located around 700 m from the entrance and approximately 50 m deep from the ground surface. The speleothem contained guano spots, with associated troglobitic springtails and isopods. Three nymphs, possibly *Mesodiplatysfalcifer* sp. n., were also found near the holotype collection site.

Two aspects of the position of this specimen within the cave are noteworthy. First, most earwigs observed in Brazilian caves have been found near the entrances, with rare exceptions such as *Euborelliajaneirensis* (Dohrn, 1864), which was observed near guano piles in the aphotic zone of a sandstone cave (Kamimura and Ferreira 2017). Thus, it is uncommon for this group to occur in deeper zones of caves in Brazil. Second, the presence of possible nymphs of this species indicates an established population rather than occasional use of the cave.

Organisms that live in subterranean environments are frequently classified into three categories (e.g., Souza-Silva et al. 2011): i) trogloxenes, which are occasionally found in caves or use caves as night-time or daytime shelters, ii) troglophiles, which can complete their entire life cycle inside or outside caves, and iii) troglobites, which do not occur in epigean habitats. Troglobites, especially those recently adapted to cave life, do not necessarily possess highly modified morphology (troglomorphy) for their exclusively hypogean life. For example, the troglobitic stonefly species *Protonemouragevi* Tierno de Figueroa et López-Rodríguez, 2010 (Plecoptera: Nemouridae) has smaller eyes and longer antennae than related congeners. Nevertheless, its general appearance resembles that of related species on the surface (Tierno de Figueroa and López-Rodríguez 2010). Although *Mesodiplatysfalcifer* sp. n. possesses well-developed eyes, its appendages are relatively long considering its smaller body size than that of *M.venado*, which is putatively the most closely related species (Table 1). Furthermore, pigmentation is reduced in some body parts. Similar conditions have been reported in *Haplodiplatysmilloti* (Chopard, 1940) (Haplodiplatyidae), which has been exclusively reported from an entirely dark part of an African cave (Chopard 1940). In *Mesodiplatysfalcifer* sp. n., the developed eyes could be used to sense light, allowing it to avoid entrance zones or external environments, as has been observed in some troglobitic beetles (Bartkowiak et al. 1991; Pellegrini and Ferreira 2011). Given the extremely dry external environment in the collection area, as well as the intense deforestation that increased in the last decades, these caves represent a suitable but isolated habitat, providing more stable temperatures and higher humidity than the surrounding epigean habitats. Hence, this new species might be a troglophile like *E.janeirensis* or a troglobite endemic to caves of the region. Further samples and studies of the ecology of this new species are necessary to determine the status of this species.

### Distribution of Mesodiplatys species

*Mesodiplatysfalcifer* sp. n. is the first diplatyid species described from Brazil except for *Cylindrogaster* spp. (Cylindrogastrinae), which are characterized by simple, non-bifurcated virgae (Haas 2012; Kamimura and Ferreira 2017). In both *M.venado* and *M.falcifer* sp. n., the parameres are much shorter than the entire length of the male genitalia, and are articulated with the main body perpendicular to its anterior-posterior axis. These similarities in genital morphology strongly support the view that these two South American species form a monophyletic clade. As suggested by Anisyutkin (2014), the monophyly of the genus as a whole is also supported by multiple genital characteristics: the relatively simple structure of the parameres, which lack branching and/or accessory structures (epimeres), and the whip-like bifurcated virgae, which have very short, undivided parts. However, parallel evolution of genital structures has been reported in several insect groups (e.g., Yoshizawa et al. 2016). Molecular studies are needed to confirm the phylogenetic relationships between Malagasy and South American *Mesodiplatys* species.

Under the assumption that *Mesodiplatys* is monophyletic, the present study provides further evidence of their enigmatic disjunct distribution. From South America (Republic of Colombia and southward), only approximately 20 species belonging to Diplatyidae or Haplodiplatyidae have been reported to date (Popham 2000). Nevertheless, recent successive discoveries of *Mesodiplatys* spp. (Anisyutkin 2014; this study) strongly indicate that this region has not been sufficiently surveyed. Although far more Diplatyidae + Haplodiplatyidae species (> 35) have been reported from Africa (Popham 2000), only one cavernicolous species, *Haplodiplatysmilloti*, has been reported (Chopard 1940). Further exploratory studies of cave earwig fauna are required before it can be concluded that *Mesodiplatys* spp. are extinct in Africa or have expanded their distribution between Madagascar and South America.

### Definitions of the genus Haplodiplatys and the family Haplodiplatyidae

Although the phylogenetic relationships between dermapteran taxa remain largely unsettled, detailed studies on their morphology suggest paraphyly of Diplatyidae*sensu lato* (including Haplodiplatyidae), among which *Haplodiplatys* is one of the earliest offshoots of Neodermaptera (Haas 1995; Haas and Kukalová-Peck 2001; Haas and Klass 2003; Klass 2003). Many species of the genus *Haplodiplatys* are characterized by multiple features presumed to be plesiomorphic, including laterally symmetrical tegmina, the absence of a spiny ridge on the dorsal side of the mesothorax, and poor differentiation of the head into frontal and occipital regions (Hincks 1955; Haas 1995; Haas and Kukalová-Peck 2001). According to this view, Engel removed this genus from Diplatyidae and placed it in the monogeneric family Haplodiplatyidae (Engel et al. 2017). Engel et al. (2017) also noted that the posterior margin of the metasternum is straight in *Haplodiplatys*, but frequently concave in Diplatyidae. *Mesodiplatysfalcifer* sp. n., however, possesses well-developed spiny ridges together with laterally asymmetrical tegmina (Figs 1, 8), as do *M.venado* (Anisyutkin 2014) and *M.insularis* (Figs 16, 18). In these species, the posterior margin of the metasternum is emarginated, as is common in Diplatyidae. Thus, aside from the relatively simple structure of the parameres, which may be homoplasious, there is no reason to move *Mesodiplatys* from Diplatyidae to Haplodiplatyidae.

Steinmann (1974; 1986b) revised the taxonomy of Diplatyidae sensu lato (as Diplatyinae in Pygidicranidae) mainly based on parameres shape, a trait which is usually described and illustrated in contemporary taxonomic studies of Dermaptera. In contrast, the spiny ridges and metasternum have seldom been described in detail in previous studies. These structures should be carefully observed in future studies to stabilize the taxonomy of this group.

## Supplementary Material

XML Treatment for Mesodiplatys falcifer

## References

[B1] Ali JR Krause DW (2011) Late Cretaceous bioconnections between Indo-Madagascar and Antarctica: refutation of the Gunnerus Ridge causeway hypothesis. Journal of Biogeography 38: 1855–1872. 10.1111/j.1365-2699.2011.02546.x

[B2] Anisyutkin LN (2014) *Mesodiplatysvenado* sp. nov. (Dermaptera: Diplatyidae), probable evidence of contact between Neotropical and Malagasy faunas. Zookeys 3794: 593‒599. 10.11646/zootaxa.3794.4.11 24870348

[B3] Bartkowiak D Tscharntke T Weber F (1991) Effects of stabilizing selection in the regressive evolution of compound eyes in hypogean carabid beetles. Mémoires de Biospéologie 18: 19–24.

[B4] Borelli A (1932a) Dermaptères nouveaux du Muséum zoologique de Hamburg. Konowia 11: 87–97.

[B5] Borelli A (1932b) Dermaptères de Madagascar. Bulletin de la Société des naturalistes luxembourgeois (N Série) 26: 56–58.

[B6] Brindle A (1966) The Dermaptera of Madagascar. Transactions of the Royal Entomological Society of London 118: 221–259. 10.1111/j.1365-2311.1966.tb02305.x

[B7] Brindle A (1967) A designation of a neo-type for *Diplatysolsofiewi* Borelli (Dermaptera, Diplatyidae), with a redescription of the species. Entomologist’s Monthly Magazine 103: 73–76.

[B8] Brindle A (1969) Dermaptera. Fauna de Madagascar, Paris 30: 1–112.

[B9] Burr M (1904) Observations on the Dermatoptera, including revisions of several genera and descriptions of New Genera and Species. Transactions of the Entomological Society of London 1904: 277–322. 10.1111/j.1365-2311.1904.tb02747.x

[B10] Burr M (1911) A revision of the genus *Diplatys* Serville (Dermaptera). Transactions of the Entomological Society of London 1911: 21–47.

[B11] Burr M (1914) Notes on the Forficularia. XXIII. More new Species. Annals and Magazine of Natural History 8: 420–428. 10.1080/00222931408693597

[B12] Case JA (2002) A new biogeographical model for dispersal of late Cretaceous vertebrates into Madagascar and India. Journal of Vertebrate Paleontology 22 (3, supplement): 42A.

[B13] Chopard L (1940) Un remarquable Dermaptera cavernicole de l’Afrique occidentale, *Diplatysmilloti* sp. n. Bulletin de la Société Zoologique de France 65: 75–79.

[B14] Engel MS Huang D Thomas JC Cai C (2017) A new genus and species of pygidicranid earwigs from the Upper Cretaceous of southern Asia (Dermaptera: Pygidicranidae). Cretaceous Research 69: 178–183. 10.1016/j.cretres.2016.09.009

[B15] Haas F (1995) The phylogeny of the Forficulina, a suborder of the Dermaptera. Systematic Entomology 20: 85–98. 10.1111/j.1365-3113.1995.tb00085.x

[B16] Haas F (2012) A Ordem Dermaptera. In: Rafael JA, Melo ARG, Carvalho CJB, Casari SA, Constantino R (Eds) Insetos do Brasil: diversidade e taxonomia. Holos Editora, Sao Paulo, Brazil, 297‒305.

[B17] Haas F Klass K-D (2003) The basal phylogenetic relationships in the Dermaptera. In: Klass K-D (Ed.) Proceedings of the 1st Dresden meeting on insect phylogeny: Phylogenetic Relationships within the Insect Orders (Dresden, September 19–21, 2003).Entomologische Abhandlungen 61: 138–142. http://www.senckenberg.de/files/content/forschung/publikationen/arthropodsystematics/ea_61_2/ea_61_2-119-172_klass.pdf

[B18] Haas F Kukalová-Peck J (2001) Dermaptera hindwing structure and folding: New evidence for familial, ordinal and superordinal relationships within Neoptera (Insecta). European Journal of Entomology 98: 445–509. 10.14411/eje.2001.065

[B19] Hadley A (2008) *Combine ZM imaging software.* Available from: https://combinezm.en.lo4d.com/details [Accessed 10 February 2018]

[B20] Hincks WD (1953) Liste préliminaire des Dermapteres de Madagascar. Mémoires de l’Institut Scientifique de Madagascar (E) 4: 361–382.

[B21] Hincks WD (1955) A Systematic Monograph of the Dermaptera of the World. Part I. Pygidicranidae. Subfamily Diplatyinae. British Museum (Natural History), London, 132 pp.

[B22] Hincks WD (1957) New species of the genus *Diplatys* Serville (Dermaptera: Pygidicranidae). Proceedings of the Royal Entomological Society of London B 26: 149–154.

[B23] Kamimura Y (2014) Pre- and postcopulatory sexual selection and the evolution of sexually dimorphic traits in earwigs (Dermaptera). Entomological Science 17: 139–166. 10.1111/ens.12058

[B24] Kamimura Y Ferreira RL (2017) Earwigs from Brazilian caves, with notes on the taxonomic and nomenclatural problems of the Dermaptera (Insecta). ZooKeys 713: 25–52. 10.3897/zookeys.713.15118 PMC5704199 29187791

[B25] Klass K-D (2003) The female genitalic region in basal earwigs (Insecta: Dermaptera: Pygidicranidae s.l.). Entomologische Abhandlungen 61: 173–225. http://www.senckenberg.de/files/content/forschung/publikationen/arthropodsystematics/ea_61_2/ea_61_2-173-225_klass.pdf

[B26] Noonan BP Chippindale PT (2006a) Dispersal and vicariance: The complex evolutionary history of boid snakes. Molecular Phylogenetics and Evolution 40: 347–358. 10.1016/j.ympev.2006.03.010 16624591

[B27] Noonan BP Chippindale PT (2006b) Vicariant origin of Malagasy reptiles supports Late Cretaceous Antarctic land bridge. The American Naturalist 168: 730–741. 10.1086/509052 17109316

[B28] Pellegrini TG Ferreira RL (2011) *Coarazuphiumtapiaguassu* (Coleoptera: Carabidae: Zuphiini), a new Brazilian troglobitic beetle, with ultrastructural analysis and ecological considerations. Zootaxa 3116: 47–58. 10.11646/zootaxa.3765.6.2 24870919

[B29] Popham EJ (2000) The geographical distribution of the Dermaptera (Insecta) with reference to continental drift. Journal of Natural History 34: 2007–2027. 10.1080/00222930050144837

[B30] Sakai S (1996) Dermapterorum Catalogus XXXI: Notes on contemporary classification of Dermaptera and recent references of Dermaptera. Daito Bunka University, Tokyo, 588 pp.

[B31] Souza-Silva M Martins RP Ferreira RL (2011) Cave lithology determining the structure of the invertebrate communities in the Brazilian Atlantic Rain Forest. Biodiversity and Conservation 20: 1713–1729. 10.1007/s10531-011-0057-5

[B32] Steinmann H (1974) A new generic classification of the species group of *Diplatys* Serville (Dermaptera: Pygidicranidae). Acta Zoologica Academiae Scientiarum Hungaricae 20: 187–205.

[B33] Steinmann H (1986a) Dermaptera. Catadermaptera I. Das Tierreich 102: 1–343.

[B34] Steinmann H (1986b) A new generic classification for the *Diplatys* species-groups (Dermaptera: Pygidicranidae). Acta Zoologica Hungarica 32: 169–179.

[B35] Tierno de Figueroa JM López-Rodríguez MJ (2010) *Protonemuragevi* sp. n., a cavernicolous new species of stonefly (Insecta: Plecoptera). Zootaxa 2365: 48–54.

[B36] Yoshizawa K Yao I Lienhard C (2016) Molecular phylogeny reveals genital convergences and reversals in the barklouse genus *Trichadenotecnum* (Insecta: Psocodea: ‘Psocoptera’: Psocidae). Molecular Phylogenetics and Evolution 94: 358–364. 26435003 10.1016/j.ympev.2015.09.018

